# Glycaemic index: did Health Canada get it wrong? Position from the International Carbohydrate Quality Consortium (ICQC)

**DOI:** 10.1017/S0007114513003905

**Published:** 2014-01-28

**Authors:** International Carbohydrate Quality Consortium, David J. A. Jenkins, Walter C. Willett, Arne Astrup, Livia S. A. Augustin, Sara Baer-Sinnott, Alan W. Barclay, Inger Björck, Jennie C. Brand-Miller, Furio Brighenti, Anette E. Buyken, Antonio Ceriello, Cyril W. C. Kendall, Carlo La Vecchia, Geoffrey Livesey, Simin Liu, Andrea Poli, Gabriele Riccardi, Salwa W. Rizkalla, John L. Sievenpiper, Antonia Trichopoulou, Thomas M. S. Wolever

**Affiliations:** Department of Nutritional Sciences, Faculty of Medicine, University of Toronto, Clinical Nutrition and Risk Factor Modification Centre, St Michael's Hospital, Toronto, Ontario, Canada; Department of Nutrition, Harvard School of Public Health, Boston, MA, USA; Department of Nutrition, Exercise and Sports (NEXS), Faculty of Science, University of Copenhagen, Copenhagen, Denmark; Clinical Nutrition and Risk Factor Modification CentreSt Michael's Hospital, Toronto, OntarioCanada; Oldways, Boston, MA, USA; Australian Diabetes Council, Glycemic Index Foundation, Sydney, Australia; Antidiabetic Food Centre, Lund University, Lund, Sweden; Boden Institute of Obesity, Nutrition, Exercise and Eating Disorders, University of Sydney, Sydney, Australia; Department of Food Science, University of Parma, Parma, Italy; Department of Nutritional Epidemiology, University of Bonn, Bonn, Germany; Institut d'Investigacions Biomëdiques August Pi i Sunyer (IDIBAPS), Barcelona, Spain; Department of Nutritional Sciences, Faculty of Medicine, University of Toronto, Toronto, Ontario, Canada; College of Pharmacy and Nutrition, University of Saskatchewan, Saskatoon, Canada; Department of Epidemiology, Mario Negri Institute, and Professor of Epidemiology, University of Milan, Milan, Italy; Independent Nutrition Logic, Wymondham, UK; Department of Epidemiology and Medicine, Brown University, Providence, RI, USA; Nutrition Foundation of Italy, Milan, Italy; Department of Clinical Medicine and Surgery, Federico II University, Naples, Italy; National Institute of Health and Medical Research (INSERM), ICAN Institute of Cardiometabolism & Nutrition, University Pierre et Marie Curie – Paris 6, Centre of Research in Human Nutrition, Pitié Salpêtrière Hospital, Paris, France; Toronto 3D Knowledge Synthesis and Clinical Trials Unit, Clinical Nutrition and Risk Factor Modification Centre, St Michael's Hospital, Toronto, Ontario, Canada; Department of Pathology and Molecular Medicine, Faculty of Health Sciences, McMaster University, Hamilton, Ontario, Canada; World Health Organization Collaborating Centre for Food & Nutrition, Department of Hygiene and Epidemiology, University of Athens Medical School, Hellenic Health Foundation, Athens, Greece; Department of Nutritional Sciences, University of Toronto, Toronto, Ontario, Canada

On behalf of Health Canada, Aziz *et al.* ^(^ ^1^ ^)^ recently published their evaluation of the use of glycaemic index (GI) claims on food labels. Although the importance of controlling postprandial glycaemia (PPG) was recognised in the position statement, they expressed the view that the GI could be ‘misleading’ and ‘would not add value’ to the existing standards for nutrition labels. Unfortunately, several statements indicate a lack of understanding of the evidence base for current information on food labels and of the GI concept in particular.

The clinical relevance of PPG is now recognised by health institutions worldwide^(^ ^2^ ^,^ ^3^ ^)^. Ideally, plasma glucose levels at the 2 h time point after a meal should be < 7·8 mmol/l since values above this level are considered to indicate the presence of impaired glucose tolerance (IGT), which may be indicative of pre-diabetes, a condition which is more prevalent than diabetes itself. Both type 2 diabetes mellitus and IGT are increasing at an alarming rate, largely due to obesity and sedentary lifestyles. Mitigating the risk of adverse outcomes associated with elevated PPG is an important target for population health.

For food labelling purposes, the challenge is to find the best tool for evaluating a product's impact on PPG within the context of other health recommendations. Although the GI has a long history of use in research and clinical practice, Aziz *et al.* ^(^ ^1^ ^)^ concluded that the GI was not useful because: (1) it has poor accuracy and precision for labelling purposes, (2) it does not vary in response to the amount of food consumed and (3) it is not congruent with national nutritional policies and guidelines.

To address the first issue, the GI methodology is recognised and described by the International Standards Organization (26 642:2010) and by the Food and Agriculture Organization of the United Nations^(^ ^4^ ^)^ as a method to assess the glycaemic impact of available carbohydrates. The GI value of one food is calculated from 640 data points (ten subjects, eight blood samples, in duplicate, one test series for the test food and three test series for the reference food). The margin of error of < 15 % (i.e. the standard error of the mean expressed as a percentage of the mean) is considered reliable in the context of the considerable day-to-day variation in glucose tolerance in healthy individuals ( ± 30–50 %)^(^ ^5^ ^)^. By testing a reference food, the GI method takes into account ‘between-person variation’.

Concerning the accuracy and precision of any nutritional attribute, one cannot let perfect be the enemy of good. For example, both whole-grain and fibre claims are permitted on food labels, despite the fact that the definition and measurement of each varies among nations and is neither perfect nor precise. A whole-grain product may contain only 50 % whole grains according to the Food and Drug Administration, and there is marked disagreement of what fibre is and how it should be measured. Moreover, total carbohydrates on food labels are often described as ‘carbohydrate by difference’, which is calculated by subtracting the sum of the water, protein, fat, dietary fibre, ash and alcohol contents from 100. This method compounds the errors associated with all assays and often differs markedly from the direct measurement of the available carbohydrate. In addition, there is a permitted margin of error of < 20 % for any component listed in the nutrition panel, which is considerably higher than the margin of error considered reliable for the GI of a food ( < 15 %). In this context, the GI is being held to a much higher standard than other nutritional attributes.

The second issue identified by Aziz *et al.* ^(^ ^1^ ^)^ was that the GI does not vary in response to the amount of food consumed. Informed consumers would anticipate that the greater the amount of the available carbohydrate consumed, the greater the increase in blood glucose. The key value of the GI therefore is that it allows comparisons between foods on a gram-for-gram carbohydrate basis, which is important for consumer choice. The glycaemic load (GL) per serving (the product of the available carbohydrate content × GI) varies in response to the amount consumed^(^ ^6^ ^)^, and could be included in the nutrient panel together with the GI.

With respect to the third issue, Health Canada claims that the GI is not congruent with national nutritional policies and guidelines, implying that the GI would be used in isolation, irrespective of other important attributes such as saturated fat, fibre and whole grain content. We agree that the GI should not override sound dietary advice^(^ ^1^ ^)^. However, this concern relates to any dietary claim, including ‘low fat’ and ‘high fibre’. Of note, Health Canada's concern is inconsistent with their earlier statement that ‘low-GI diets have attributes of generally recognized healthy eating patterns’^(^ ^1^ ^)^. However, to address their concern that the composition of a low-GI food may not always be congruent with nutritional guidelines, our suggestion would be to consider a GI claim in conjunction with a healthy food profile. Programmes such as the GI symbol in Australia require the fulfilment of strict nutritional criteria that are consistent with dietary guidelines in order for a food to be eligible to use the certified GI logo.

We agree with Aziz *et al.* ^(^ ^1^ ^)^ that ‘consumers are familiar with the concept, even though their understanding of it might not be accurate’. In our view, this largely reflects the lack of communication about the GI to the general public and health professionals. The assumption that the GI concept may be too difficult for the lay person is not supported by the Australian experience, where surveys indicate that one in four Australians look for healthy low-GI foods when shopping, simply substituting healthy low-GI varieties for regular high-GI variants within a food group/category^(^ ^7^ ^)^. Moreover, low-GI dietary advice in randomised clinical trials is associated with high completion rates (low attrition), suggesting that simple low-GI communications can be effective^(^ ^8^ ^,^ ^9^ ^)^. As in the case of quality of fat (saturated, monounsaturated and polyunsaturated), health agency advice preceded information now commonly listed in the nutritional panel^(^ ^10^ ^)^.

Finally, in their conclusions, Aziz *et al.* ^(^ ^1^ ^)^ proposed that nutritional recommendations should take a food-based approach. We agree, yet Health Canada's recommendation to increase intakes of whole foods in the form of vegetables, fruits, grains and pulses does not address the main carbohydrate sources of most populations, i.e. breads, breakfast cereals, rice and ready-to-eat cereal products. Pasta, a staple carbohydrate food of the heart-healthy Mediterranean diet, is a refined yet low-GI carbohydrate food. Most basmati and parboiled rice are white yet have a low GI. There is also a need to distinguish high-GI from low-GI whole grains. Indeed, advice to ‘choose more intact, unprocessed or minimally processed whole-grain products instead of their highly processed counterparts’ is aimed at lowering overall dietary GI or GL. It is a common myth that all whole-grain products have low-GI values when in fact many are highly processed and correspondingly easy to digest^(^ ^11^ ^)^. In clinical trials, low-GI diets have produced superior outcomes compared with the high-fibre–high-GI diets^(^ ^8^ ^,^ ^9^ ^,^ ^12^ ^)^. We suggest that GI labels may in fact stimulate the food industry to produce genuinely healthier whole-grain products that retain the low GI of the original grain.

Finally, if GI values are misleading and unreliable as Health Canada claims, then it is truly remarkable that a lower dietary GI/GL has been independently associated with a reduced risk of type 2 diabetes^(^ ^13^ ^)^ and cardiovascular disease^(^ ^14^ ^)^ in large prospective cohort studies of diverse populations^(^ ^15^ ^)^. Similarly, randomised controlled trials have shown the benefits of low-GI diets for weight management^(^ ^8^ ^,^ ^9^ ^,^ ^12^ ^)^, serum lipids^(^ ^9^ ^,^ ^12^ ^,^ ^16^ ^)^, insulin sensitivity^(^ ^17^ ^)^ and inflammatory markers^(^ ^18^ ^)^. Most importantly, the selection of low-GI foods has resulted in the successful improvements of glycaemic control, dyslipidaemia and inflammation in people with type 2 diabetes^(^ ^9^ ^,^ ^18^ ^,^ ^19^ ^)^. In this regard, these lines of evidence have been used to support the inclusion of low-GI and low-GL dietary patterns in the evidence-based nutrition recommendations of the Canadian Diabetes Association, American Diabetes Association, Diabetes UK, Diabetes Australia, International Diabetes Federation and the European Association for the Study of Diabetes^(^ ^20^ ^)^. If GI values were not precise, one would not expect to see distinct differences in PPG in response to low- or high-GI meals observed at different time points throughout the day^(^ ^12^ ^)^. These beneficial outcomes would not be possible if the GI concept were unduly undermined by large variability or differences among people of different ethnicity.

Taken together, Health Canada's evaluation misinterprets and misrepresents current scientific evidence, in part by taking the GI outside the context of a healthy diet. In view of the proven health benefits of low-GI diets ‘as currently defined and measured’, every effort should be made to assist consumers in choosing carbohydrate foods that will not exacerbate PPG.

## Authors

*International Carbohydrate Quality Consortium (ICQC)*

*David J. A. Jenkins (ICQC chair)*, Department of Nutritional Sciences, Faculty of Medicine, University of Toronto, Clinical Nutrition and Risk Factor Modification Centre, St Michael's Hospital, Toronto, Ontario, Canada.

*Walter C. Willett (ICQC chair)*, Department of Nutrition, Harvard School of Public Health, Boston, MA, USA.

*Arne Astrup*, Department of Nutrition, Exercise and Sports (NEXS), Faculty of Science, University of Copenhagen, Copenhagen, Denmark.

*Livia S. A. Augustin*, Clinical Nutrition and Risk Factor Modification Centre, St Michael's Hospital, Toronto, Ontario, Canada.

*Sara Baer-Sinnott*, Oldways, Boston, MA, USA.

*Alan W. Barclay*, Australian Diabetes Council, Glycemic Index Foundation, Sydney, Australia.

*Inger Björck*, Antidiabetic Food Centre, Lund University, Lund, Sweden.

*Jennie C. Brand-Miller*, Boden Institute of Obesity, Nutrition, Exercise and Eating Disorders, University of Sydney, Sydney, Australia.

*Furio Brighenti*, Department of Food Science, University of Parma, Parma, Italy.

*Anette E. Buyken*, Department of Nutritional Epidemiology, University of Bonn, Bonn, Germany.

*Antonio Ceriello*, Institut d'Investigacions Biomèdiques August Pi i Sunyer (IDIBAPS), Barcelona, Spain.

*Cyril W. C. Kendall*, Department of Nutritional Sciences, Faculty of Medicine, University of Toronto, Toronto, Ontario, Canada; College of Pharmacy and Nutrition, University of Saskatchewan, Saskatoon, Canada.

*Carlo La Vecchia*, Department of Epidemiology, Mario Negri Institute, and Professor of Epidemiology, University of Milan, Milan, Italy.

*Geoffrey Livesey*, Independent Nutrition Logic, Wymondham, UK.

*Simin Liu*, Department of Epidemiology and Medicine, Brown University, Providence, RI, USA.

*Andrea Poli*, Nutrition Foundation of Italy, Milan, Italy.

*Gabriele Riccardi*, Department of Clinical Medicine and Surgery, Federico II University, Naples, Italy.

*Salwa W. Rizkalla*, National Institute of Health and Medical Research (INSERM), ICAN Institute of Cardiometabolism & Nutrition, University Pierre et Marie Curie – Paris 6, Centre of Research in Human Nutrition, Pitié Salpêtrière Hospital, Paris, France.

*John L. Sievenpiper*, Toronto 3D Knowledge Synthesis and Clinical Trials Unit, Clinical Nutrition and Risk Factor Modification Centre, St Michael's Hospital, Toronto, Ontario, Canada; Department of Pathology and Molecular Medicine, Faculty of Health Sciences, McMaster University, Hamilton, Ontario, Canada.

*Antonia Trichopoulou*, World Health Organization Collaborating Centre for Food & Nutrition, Department of Hygiene and Epidemiology, University of Athens Medical School, Hellenic Health Foundation, Athens, Greece.

*Thomas M. S. Wolever*, Department of Nutritional Sciences, University of Toronto, Toronto, Ontario, Canada.
